# Metabolite itaconate in host immunoregulation and defense

**DOI:** 10.1186/s11658-023-00503-3

**Published:** 2023-12-02

**Authors:** Wenchang Yang, Yaxin Wang, Kaixiong Tao, Ruidong Li

**Affiliations:** 1 Department of Gastrointestinal Surgery, Union Hospital, Tongji Medical College, Huazhong University of Science and Technology, No. 1277 Jiefang Avenue, Wuhan, 430022 Hubei China; 2 Department of Critical Care Medicine, Union Hospital, Tongji Medical College, Huazhong University of Science and Technology, Wuhan, 430022 China; 3 Department of Gastrointestinal Surgery, Affiliated Hospital of Qingdao University, Qingdao, China

**Keywords:** Itaconate, Itaconate derivative, Immunometabolism, Defense

## Abstract

Metabolic states greatly influence functioning and differentiation of immune cells. Regulating the metabolism of immune cells can effectively modulate the host immune response. Itaconate, an intermediate metabolite derived from the tricarboxylic acid (TCA) cycle of immune cells, is produced through the decarboxylation of cis-aconitate by cis-aconitate decarboxylase in the mitochondria. The gene encoding cis-aconitate decarboxylase is known as immune response gene 1 (*IRG1*). In response to external proinflammatory stimulation, macrophages exhibit high IRG1 expression. IRG1/itaconate inhibits succinate dehydrogenase activity, thus influencing the metabolic status of macrophages. Therefore, itaconate serves as a link between macrophage metabolism, oxidative stress, and immune response, ultimately regulating macrophage function. Studies have demonstrated that itaconate acts on various signaling pathways, including Keap1-nuclear factor E2-related factor 2-ARE pathways, ATF3–IκBζ axis, and the stimulator of interferon genes (STING) pathway to exert antiinflammatory and antioxidant effects. Furthermore, several studies have reported that itaconate affects cancer occurrence and development through diverse signaling pathways. In this paper, we provide a comprehensive review of the role IRG1/itaconate and its derivatives in the regulation of macrophage metabolism and functions. By furthering our understanding of itaconate, we intend to shed light on its potential for treating inflammatory diseases and offer new insights in this field.

## Introduction

Macrophages, which are critical components of innate immunity, can be activated by various infections or tissue damage [1, 2]. Upon activation, macrophages undergo metabolic reprogramming to meet the increased biosynthetic demands [3, 4]. The tricarboxylic acid (TCA) cycle serves as the primary metabolic pathway for eukaryotes to generate energy [5]. Metabolites of the TCA cycle reprogram the metabolism and epigenetics of immune cells, enabling them to perform different functions [6, 7]. Immune responsive gene 1 (*IRG1*), which encodes cis-aconitate decarboxylase (CAD), a metabolic enzyme of TCA cycle, catalyzes the decarboxylation of cis-aconitate to produce itaconate [8]. Itaconate is highly expressed in activated macrophages, which can influence metabolite and mitochondrial respiration changes in macrophages [9].

Itaconate was first discovered and obtained by Samuel Baup in 1836 [10]. In the late 1920s, Japanese scholars isolated itaconic *Aspergillus*, capable of producing itaconate, in salt-impregnated plum juice, marking the earliest discovery of a microorganism with itaconate production capability [11]. In 2011, Shin et al. [12] first reported that itaconate was only increased in *M. tuberculosis*-infected lung tissue, speculating that the itaconate in lung tissue may originate from *M. tuberculosis*. Subsequently, Strelko et al. [13] demonstrated a substantial increase in itaconate production and secretion in activated macrophages upon stimulation with lipopolysaccharide (LPS) or interferon-gamma (IFN-γ). In 2013, Michelucci et al.[14] revealed the biosynthesis pathway of itaconate in mammals. Since then, multitudinous studies have highlighted the crucial role of itaconate as a key link between immune response, metabolism, and inflammation [14, 15]. Research on itaconate and its derivatives holds great significance in the treatment of inflammatory and immune-related diseases [16–18]. Inflammatory autoimmune diseases encompass a broad range of conditions resulting from human dysfunction, including psoriasis, rheumatoid arthritis, systemic lupus erythematosus (SLE), multiple sclerosis (MS), and inflammatory bowel disease (IBD) [19]. The incidence of inflammatory diseases is on the rise due to increased exposure to physical factors, chemical factors, and biological factors, as well as lifestyle changes. Chronic inflammatory diseases have a long-term impact on the quality of life of patients. Itaconate exerts anti-inflammatory and antioxidative stress effects through diverse mechanisms and plays a key role in infectious diseases, immune-related diseases, and certain malignancies. In this study, we comprehensively examine and review the structure of itaconate, biosynthesis, metabolism, derivatives, and modulation of *IRG1* as well as the regulation of signaling pathways and progress in the treatment of different diseases related to itaconate. The insights gained from this study will serve as a theoretical foundation for further research and the development of therapeutic approaches targeting inflammatory diseases.

### The structure of itaconate and metabolism of itaconate

The molecular formula of itaconate is C_5_H_6_O_4_, with a molecular weight of 130.1 g/mol. It exists as white powdery or colorless crystals and remains stable when stored at room temperature. Itaconate is a five-carbon dicarboxylic acid with an α,β-unsaturated alkene structure [20]. Structurally and chemically, itaconate is similar to other metabolites such as phosphoenolpyruvate, succinate, and fumarate [21]. Its unsaturated double bond and two active carboxyl groups enable it to undergo various chemical reactions, with the esterification of itaconate being particularly substantial.

In 2013, Michelucci et al. [14] first elucidated the pathway of itaconate biosynthesis in mammals. Itaconate, an intermediate product of the TCA cycle, is produced through the decarboxylation of cis-aconitate by CAD, which is encoded by *IRGI*. The researchers also performed IRG1 protein purification and directly demonstrated that IRG1 catalyzes the decarboxylation of cis-aconitate. Furthermore, they observed lower levels of itaconate production in human macrophages than in mouse macrophages. Additionally, IRG1 protein expression was absent in resting macrophages, but highly expressed in activated macrophages. These characteristics of IRG1/itaconate have garnered great attention from academic researchers, leading to further exploration of the role of IRG1/itaconate.

### Itaconate derivatives and isomers

**Fig. 1:**
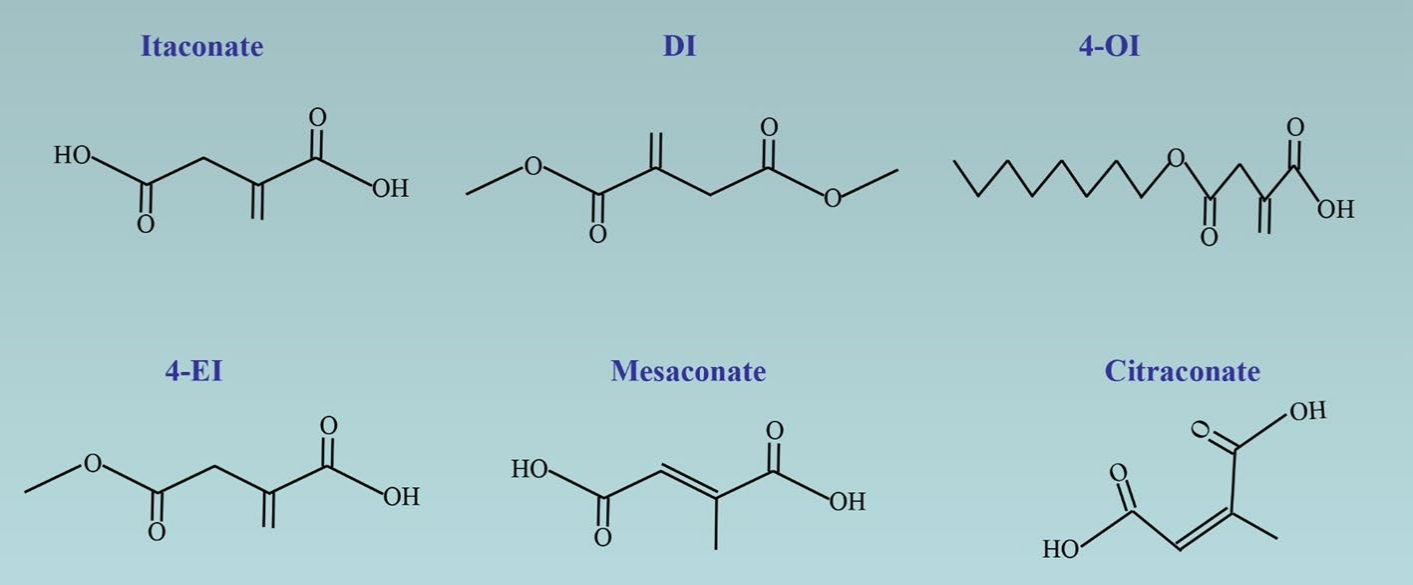
The chemical structures of itaconate, derivatives, and isomers

Notably, for itaconate to exert its anti-inflammatory effects, it needs to enter the cytoplasm. However, it is challenging for itaconate to penetrate the cell membrane in vitro. To investigate the effect of itaconate and its application in vitro and in vivo, researchers synthesized the following derivatives of itaconate: dimethyl itaconate (DI), 4-octyl itaconate (4-OI), and 4-ethyl itaconate (4-EI) (Fig. 1).

The esterification of the carboxyl group at the 1-position of DI facilitates it crossing of the cell membrane. The structural characteristics and lack of negative charge make DI easier to activate Nrf2 than itaconate [22]. Furthermore, DI activates Nrf2 target genes in a manner similar to dimethyl fumarate (DMF), another Nrf2 activator [23]. DI exhibits strong electrophilicity, which blocks LPS-induced IκBζ expression [16]. However, several studies have reported difficulties in converting DI to intracellular itaconate. ElAzzouny et al. [24] used isotopically labeled [13C]itaconate and [13C]DI and found that [13C]DI was not converted into [13C]itaconate in bone marrow-derived macrophages (BMDMs). Therefore, the metabolic effects of DI are attributed to its electrophilicity and covalent modification rather than itaconate accumulation, suggesting that itaconate derivatives are not exactly the same as itaconate. Nonetheless, they also discovered that increasing DI concentrations can enhance intracellular itaconate production in LPS-induced BMDMs. We hypothesized exogenous DI potentiates the effects of LPS activation in BMDMs, leading to increased itaconate production. However, further investigation is required to determine whether DI can be converted into itaconate.

To overcome the rapidly intracellular thiol reaction of DI, Mills et al. [25] designed a new itaconate derivative named 4-OI, where the location of the octyl ester differs from that in DI. The altered location of the octyl ester group makes 4-OI more structurally similar to intracellular itaconate. Similar to DI, Swain et al. [22] reported in 2020 that 4-OI cannot be converted into intracellular itaconate. However, in the same year, a study published in *Cell Metabolism* reported that 4-OI could directly generate itaconate [26]. They treated BMDMs with ^13^C_5_-octyl itaconate and detected the presence of ^13^C_5_-itaconate. Both DI and 4-OI exhibit strong electrophilic stress response, resulting in changes to the immunosuppressive phenotype. DI and 4-OI can inhibit the secretion of pro-IL-1β, IL-6, and IFN-β in an Nrf2-independent manner. In contrast, intracellular itaconate only suppressed IL-1β secretion but not pro-IL-1β levels [27]. Surprisingly, intracellular itaconate strongly increased the LPS-induced IFN-β production. These findings indicate that itaconate derivatives do not entirely replicate the functions of endogenous itaconate due to structural and electrophilicity changes. This discrepancy also explains why itaconate and its derivatives exhibit different or even opposing effects in some experiments.

Currently, few reports on 4-EI exist. The esterification of the 4-carboxyl in 4-EI results in lower electrophilicity compared with that of DI or 4-OI. Reportedly, 4-EI could not inhibit IκBζ activation, while the 4-EI has a consistent effect with DI on IκBζ inhibition with the addition of butylthionine sulfoxide [16, 22]. Therefore, the three derivatives differ in both structure and function. None of them can fully replicate endogenous itaconate. Further research needed to develop more refined derivatives.

Mesaconate and citraconate are isomeric derivatives of itaconate (Fig. 1). Recently, He et al. [28] demonstrated that mesaconate synthesized from itaconate inhibited glycolytic activity and the proinflammatory cytokines release. However, they found that mesaconate did not inhibit the activity of succinate dehydrogenase (SDH), suggesting that mesaconate had a lesser influence on cellular metabolism compared with itaconate. Additionally, Chen et al. [29] reported that citraconate was the strongest electrophile and nuclear factor E2-related factor 2 (Nrf2) activator compared with itaconate and mesaconate, and citraconate inhibited itaconate catalysis. These findings indicate that the isomers play different roles in certain aspects.

### Modulation of immune response gene 1

#### Toll-like receptors

**Fig. 2:**
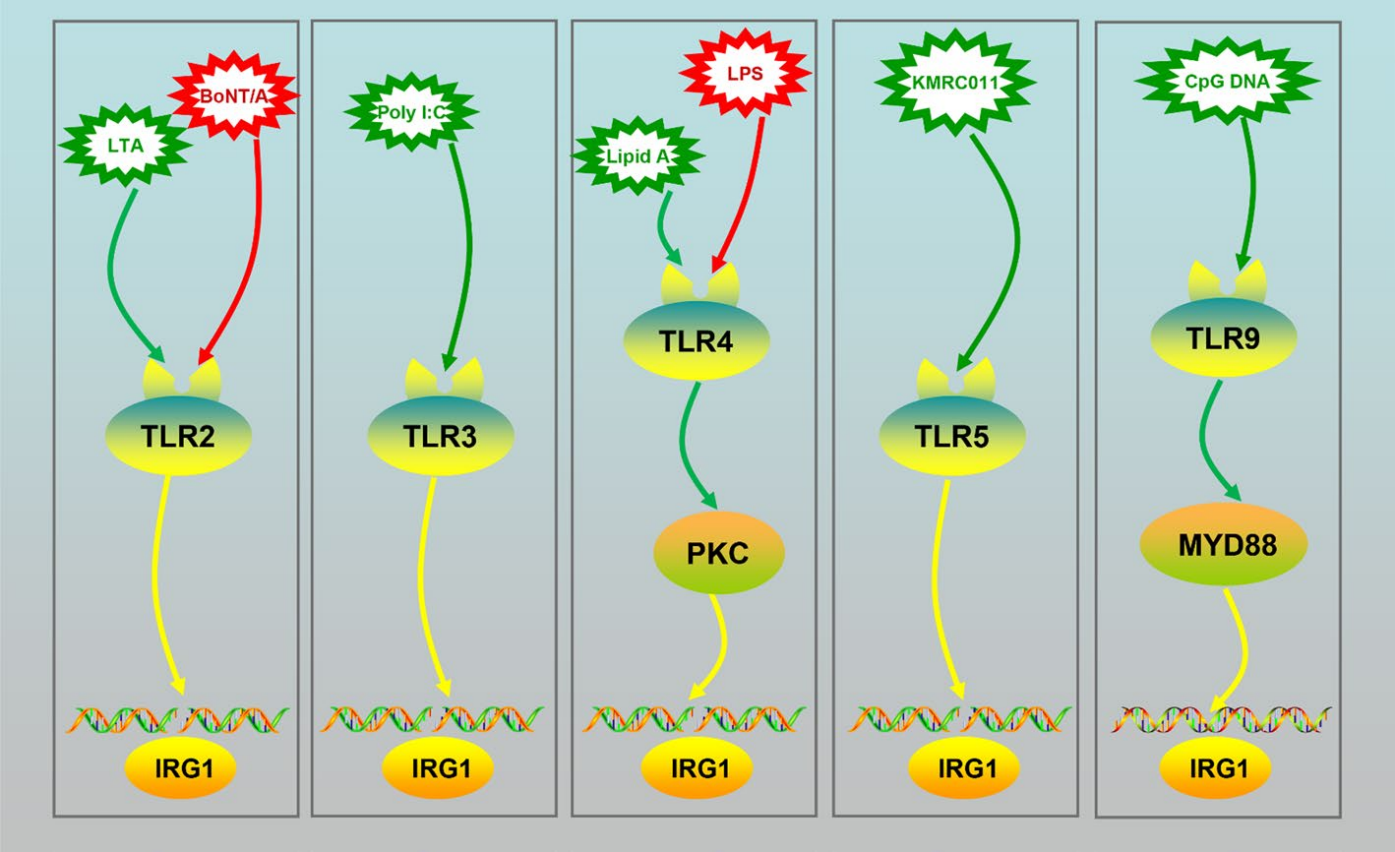
Reagents that induce IRG1 expression

Toll-like receptors (TLRs) play a crucial role in the innate immune system by recognizing invading microorganisms and activating immune cell responses [30]. Numerous studies have shown that TLRs mediate IRG1* IRG1* expression in response to various stimuli. For example, LPS and lipid A promote IRG1 expression by acting on TLR4 in primary immune cells and cell lines [31, 32]. TLR2 is required for lipoteichoic acid (LTAl)- or botulinum neurotoxin type A (BoNT/A)-induced *IRG1* mRNA expression. TLR4 or TLR2 depletion inhibited IRG1 expression in activated immune cells [33, 34]. Furthermore, TLR9 is responsible for CpG-DNA-induced *IRG1* mRNA expression [31]. Importantly, a high concentration of poly (I:C), rather than a low concentration, promoted *IRG1* expression through TLR3 in macrophages [25, 31]. Additionally, KMRC011, a TLR5 activator, increased IRG1 mRNA expression in the small intestine [35]. Myeloid differentiation primary response 88 (MYD88) is a key signaling molecule in the TLR pathway and plays an important role in transmitting upstream information [36, 37]. TLR9 signaling-medicated *IRG1* expression is not activated in Myd88^−/−^ macrophages [37]. Rodriguez et al. [38] reported that IRG1 was not upregulated in Myd88^−/−^ macrophages in *Chlamydia pneumoniae* infection, whereas it was increased in wild type (WT) macrophages, suggesting that MYD88-mediated* IRG1* expression (Fig. 2).

#### Inflammatory cytokines

Inflammatory cytokines can also induce IRG1 expression. Studies have shown that interferon beta (IFNβ) and IFNγ treatment increases *IRG1* mRNA expression in ANA-1 cells [39]. Simultaneously, Lee et al. [40] reported that type I interferon increased the expression of IRG1 while inhibiting isocitrate dehydrogenase activity. Souza et al. [41] revealed that autocrine IFN-I also increased the expression of IRG1. Furthermore, Mills et al. [25] reported that IFNβ enhanced LPS-induced IRG1 mRNA expression, which could be inhibited by depletion of the type I interferon receptor, suggesting a synergistic effect between LPS and IFNβ in mediating IRG1 expression. Additionally, Degrandi et al. [31] reported that TNF enhanced *IRG1* mRNA expression in murine ANA-1 macrophages. Overall, cytokines play a positive role in promoting IRG1 expression.

A20, a TNF-α-inducible protein, can protect cells from cytotoxicity by inhibiting the activation of inflammatory pathways [42]. Overexpression of A20 inhibited TNF-α-induced apoptosis and nuclear factor-kappaB (NF-κB) pathway activation [43]. Li et al. [44] found that the IRG1 upregulation was necessary for the upregulation of A20 via reactive oxygen species (ROS) in LPS-tolerized macrophages. Small interfering RNA targeting A20 eliminated the inhibitory effect of IRG1 on proinflammatory cytokine production. However, Quickelberghe et al. [45] reported that IRG1 was up-regulated in the absence of A20 in BMDMs, as observed in proteomics study, indicating that A20 negatively regulated IRG1 expression. Thus, further research is needed to understand the regulatory relationship between A20 and IRG1, as there appears to be a feedback and a negative feedback relationship between them.

#### Protein kinase C

Protein kinase C (PKC) is a cytoplasmic enzyme that, upon activation by second messengers, becomes a membrane-bound enzyme and can activate enzymes in the cytoplasm and participate in the regulation of biochemical reactions. Concurrently, PKC can act on transcription factors in the nucleus and contribute to gene expression regulation. Lee et al. [46] reported that activating the PKC pathway further increased LPS-induced *IRG1* mRNA expression in macrophages. However, unlike PKC, the protein kinase A did not affect the expression of IRG1.

#### miRNAs

miRNA is a small RNA molecule (21 to 23 nucleotides long) widely found in eukaryotes that regulates the expression of other genes [47]. miRNA can inhibit posttranscriptional gene expression by specifically binding to target messenger RNA (mRNA). Shi et al. [48] reported that miR-378 directly targeted and downregulated the expression of IRG1 in glioma, as validated through a luciferase reporter assay, suggesting that posttranscriptional mechanisms also regulated *IRG1* gene expression. Additionally, Azzimato et al. [49] reported that miR-144 negatively regulated IRG1 expression in obesity.

#### Others

Recently, Schuster et al. [50] reported that transcription factor EB (TFEB), a lysosomal biogenesis factor, increased the transcription of IRG1 and promoted itaconate synthesis. Li et al. [51] reported that hypoxia-inducible factor-1α (HIF-1α) transcriptionally upregulated IRG1 for itaconate synthesis. Moreover, studies have shown that viruses can promote the expression of IRG1 through specific mechanisms. Daniels et al. [52]. found that Zika virus infection activated ZBP1, RIPK1, and RIPK3, ultimately leading to IRG1 upregulation, and thereby enhancing our understanding of IRG1. These findings indicate that the body can activate IRG1 through different pathways to confer resistance against external stimuli, highlighting the diversity and complexity of immune regulation and metabolism.

### The roles of IRG1/itaconate in different signaling pathways (Fig. 3)

**Fig. 3:**
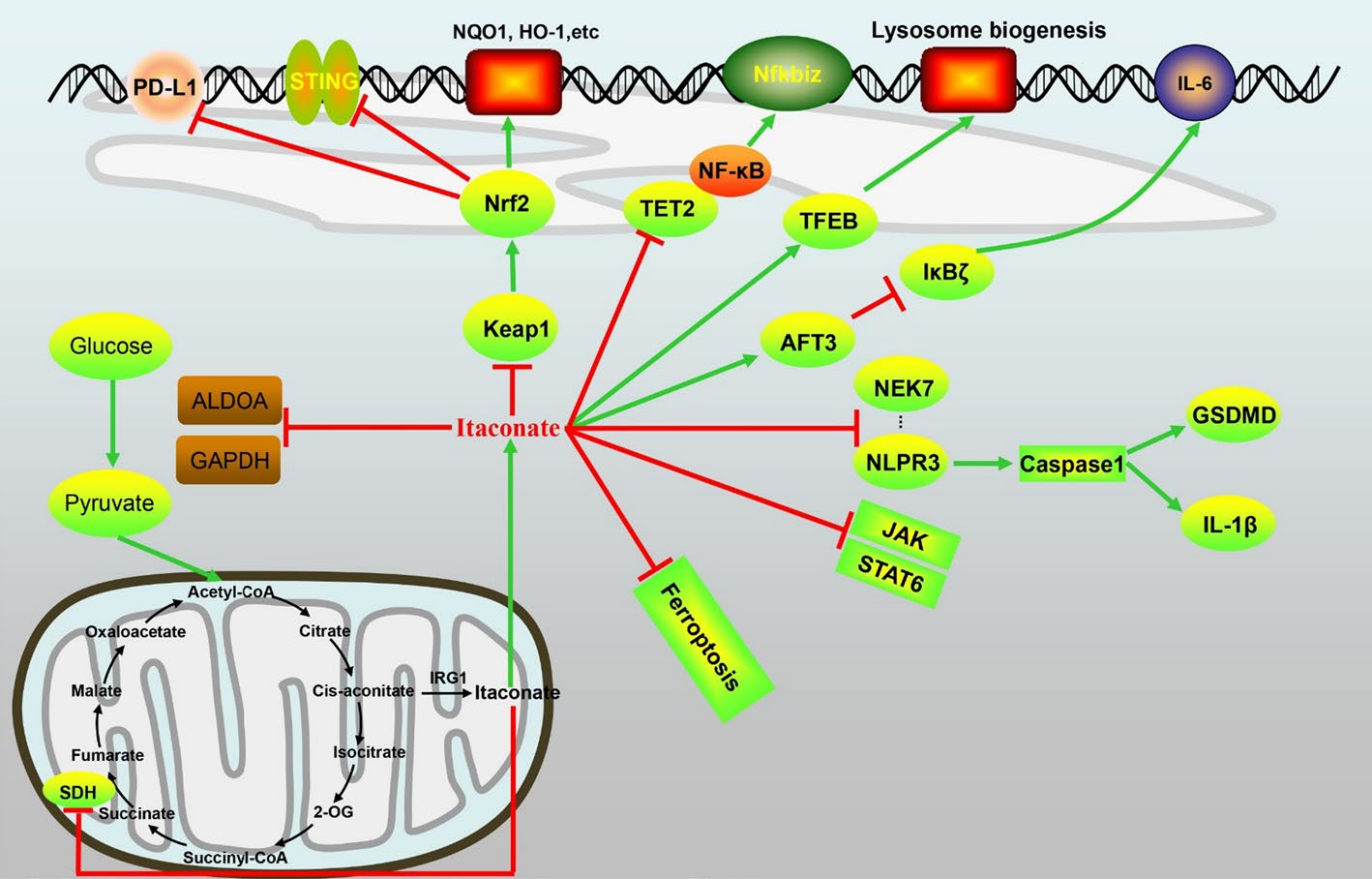
The role of itaconate in regulating signal pathways

#### Itaconate inhibited the activity of succinate dehydrogenase

Succinate dehydrogenase (SDH), a mitochondrial membrane enzyme, plays a central role in connecting oxidative phosphorylation and electron transfer; it converts succinate to fumarate and eventually to malate [53]. Accumulation of succinate resulting from SDH oxidation can promote the excessive production of reducing coenzyme Q, leading to the generation of mitochondrial ROS (mtROS) and inhibition of IL-10 expression [54, 55]. Reportedly, itaconate can inhibit mtROS production by inhibiting SHD activity [29, 56]. In 1949, Ackermann et al. [57] first reported the inhibitory effect of itaconate on SDH activity. Subsequently, Lampropoulou et al. [58] demonstrated in vitro and in vivo that itaconate inhibited the inflammatory response by impeding SDH-mediated oxidation of succinate. This inhibition occurs because the structure of itaconate is similar to that of succinate, allowing it to competitively block the active site of SDH. Additionally, itaconate inhibited Zika virus genome replication by inhibiting SDH activity [52], indicating its potential to hinder the proliferation of certain viruses. Notably, excessive inhibition of SDH by itaconate can cause immune paralysis, which can be counteracted by β-glucan. These results suggest that the IRG1–itaconate–SDH axis is not simply an inflammatory inhibition pathway but rather an immunomodulatory pathway.

#### Glycolytic inhibitor

In recent studies, itaconate and its derivatives have been suggested to exert anti-inflammatory functions through the regulation of aerobic glycolysis. Both aldolase A (ALDOA) and glyceraldehyde-3-phosphate dehydrogenase (GAPDH) enzymes, which are involved in glycolysis, possess alkylated cysteine residues [59, 60]. Qin et al. [61] reported that itaconate decreased ALDOA enzyme activity and lactate production in macrophages. Furthermore, they found a significant increase in ALDOA activity in IRG1^–/–^ macrophages. GAPDH is a rate-limiting enzyme in aerobic glycolysis, catalyzing the phosphorylation and oxidation of glyceraldehyde-3-phosphate to form 1,3-diphosphoglycerate in the presence of NAD^+^ and phosphoric acid. In 2008, Blatnik et al. [62] reported that fumarate inhibited GAPDH activity and thereby attenuated inflammation progression in diabetes. Subsequently, Kornberg et al. [63] demonstrated that DMF inactivated the catalytic cysteine of GAPDH to modulate immunity in vitro and in vivo. Given the structural similarity in α,β-putian structures between fumarate and itaconate, itaconate derivatives were speculated to target GAPDH to exert similar effects. In 2019, Liao et al. [64] reported that 4-OI directly alkylated cysteine residue 22 on the GAPDH enzyme and decreased its activity. They also observed a significant increase in GAPDH enzyme activity, lactate production, and extracellular acidification rate in IRG1^–/–^ BMDMs. Additionally, itaconate was demonstrated to inhibit the activity of fructose-6-phosphate 2-kinase [65]. These studies suggest that itaconate derivative 4-OI acts as an anti-inflammatory agent by inhibiting the activity of enzymes involved in glycolysis. This also suggests that 4-OI might play an antitumor role by inhibiting glycolysis. However, no relevant reports exist on this matter, and further research is required in the future.

#### Itaconate activates Keap1/Nrf2

The Keap1- Nrf2-antioxidant response elements (AREs) signaling pathway is a key pathway in cellular oxidative stress response. Its downstream phase II metabolic enzymes and antioxidant proteins play an important role in resisting external stimuli [66, 67]. Nrf2, a transcription factor, is crucial for the oxidative stress response and binds to AREs located in the promoter region of protective genes. Under physiological conditions, Keap1 binds to Nrf2 in the cytoplasm. When cells receive certain stimuli, Keap1–Nrf2 binding is unstable, leading to the release of Nrf2. It is then transferred to the nucleus, where it binds to AREs in the form of NrF2–Maf and activates downstream genes, including NAD(P)H:quinone oxidoreductase 1 (NQO1), glutamate cysteine ligase, and HO-1 [68, 69]. HO-1 can decompose heme into antioxidant biliverdin [70]. NQO1 can catalyze the reduction of quinones and their derivatives, degrading their toxicity and preventing ROS generation [71].

In 1990, Professor Sohal first proposed the concept of oxidative stress [72]. When the body is exposed to various harmful stimuli, excessive ROS and nitrogen radicals are produced, leading to the activation of various inflammatory pathways such as NF-κB, JAK, STAT, and MAPK. This, in turn, results in increased cytokines release and contributes to the occurrence and development of diseases [73, 74]. Itaconate has been demonstrated to exert excellent anti-inflammatory and antioxidant effects through the activation of Nrf2 [75, 76]. Mills et al. [25] found that itaconate activated Nrf2 by alkylating Keap1 cysteine residues through a Michael addition (Cys151, Cys257, Cys288), resulting in increased expression of downstream genes involved in antioxidant and anti-inflammatory processes. This effect may be attributed to the electrophilic α,β-unsaturated carboxylic acid of itaconate. Furthermore, Kobayashi et al. [77] reported that Nrf2 directly binds to the proinflammatory genes IL-6 and IL-1β in macrophages, exerting anti-inflammatory effects. In addition to its antibacterial effects, Olagnier et al. [78] reported that 4-OI exhibited potent antiviral activity against SARS-CoV2 through Nrf2 activation.

However, a study reported that high concentrations of Nrf2 activators, including 4-OI induced cell death, increased IL-1β cleavage independently of the classical inflammasome pathway [79]. The addition of pan-caspase inhibitor Q-VD-OPh in bone marrow-derived dendritic cells (BMDCs) completely abolished 4-OI-induced IL-1β release. Furthermore, itaconate-medicated apoptosis was found to be characterized by the loss of mitochondrial membrane potential leading to caspase-3-dependent caspase-8 activation. These findings further suggest that IRG1/itaconate pathway is not simply an immunosuppressive pathway but rather an immunoregulatory pathway.

### Itaconate regulates the activating transcription factor 3-IκBζ axis

Activating transcription factor 3(ATF3) is an anti-inflammatory transcription factor that not only regulates the production of cytokines such as IL-6, but is also associated with mitochondrial stress [80]. IκBζ is a nuclear protein encoded by the *NFKBIZ* gene that controls IL-6 release [81]. Under normal conditions, ATF3 activity is inhibited by IκBζ. Itaconate and its derivative DI have been shown to upregulate ATF3 and inhibit IκBζ, thereby suppressing the expression of the proinflammatory factor IL-6 [16]. Notably, the anti-inflammatory pathways of itaconate targeting the ATF3-IκBζ axis were independent of Nrf2. TNF-α is produced in the primary transcriptional response to TLR stimulation, whereas IL-6 is induced during the secondary transcriptional responses. IκBζ mainly regulates the secondary transcriptional response to TLR activation. Therefore, DI does not prevent LPS-mediated p65 nuclear translocation and TNF-α release. Consequently, the ATF3–IκBζ axis mainly inhibits the secondary transcriptional response, thereby exerting an anti-inflammatory response.

### Itaconate negatively regulates the stimulator of interferon genes pathway

The stimulator of interferon genes (STING) pathway is a unique pathway that can stimulate the body’s immune system [82]. STING activation can occur through cyclic GMP–AMP synthase (cGAS) dependent and non-cGAS-dependent forms. In the cGAS–STING pathway, cGAMP causes STING conformational changes and activation of STING, leading to its transfer from the endoplasmic reticulum to the Golgi apparatus. This results in the recruitment of TANK binding kinase 1 (TBK1) and IκB kinase (IKK) recruitment [83, 84]. Activated TBK1 and IKK then phosphorylate downstream interferon regulator IRF3 and NF-κB inhibitory protein IκBα. The activation of IRF3 and NF-κB allows them to enter into the nucleus and induce the expression of related cytokines and proteins such as IFN-γ, IL-6, and TNF-α, leading to an inflammatory response. Reportedly, treatment with 4-OI treatment significantly impaired STING protein expression and STING-dependent signaling pathway through Nrf2 activation while inhibiting the release of type I IFN [85]. Further research demonstrated that the knockdown of Nrf2 increased STING expression, TBK1 phosphorylation, and IFNB1 gene expression. Furthermore, the knockdown of Keap1 decreased STING expression. These results indicated that itaconate and 4-OI may inhibit the STING pathway through Nrf2 activation. Recently, Li et al. [86] reported that 4-OI could alkylate cysteine sites 65, 71, 88, and 147 of STING to inhibit inflammation response, which broadened the knowledge on the effect of the itaconate in immunomodulation.

### Itaconate inhibits JAK1 activation

Macrophages can be classified into two subtypes: classically activated M1 macrophages and selectively activated M2 macrophages [87]. M1 macrophages are typically activated by IFN-γ and LPS [88]. They primarily release proinflammatory factors and play an important role in the early stage of inflammation. By contrast, M2 macrophages are activated by Th2 cytokines such as IL-4 and IL-13 [89]. They express anti-inflammatory factors and contribute to the inhibition of inflammatory response and tissue repair. Itaconate and its derivative have a significant inhibitory effect on the inflammatory response in M1 macrophages [90]. Recently, Runtsch et al. [91] reported that both itaconate and 4-OI inhibited JAK1 and STAT6 phosphorylation in M2 macrophages. The itaconate derivatives directly modified the structure of JAK1, specifically cysteines 715, 816, 943, and 1130. In a model of steroid-resistant asthma, they demonstrated the suppression of M2 polarization and JAK1 activation by 4-OI, revealing a new mechanism of itaconate in immunoregulation. Blanco et al. [92] reported that 4-OI inhibited JAK1 activation and reduced the severity of murine lupus. Based on these findings,4-OI holds promise as a potential treatment strategy for diseases driven by M2 macrophages through the inhibition of JAK1.

### Itaconate inhibits TET2

As mentioned previously, itaconate can exert anti-inflammatory effects by inhibiting SDH and IκBζ and activating Keap1/Nrf2 pathways. Recently, Chen et al. [93] revealed that itaconate also regulated gene transcription and bound directly to Ten-Eleven Translocation-2 (TET2) in a manner similar to α-KG. The researchers compared the transcriptome changes between Tet2-WT with 4-OI and TET2 deletion in LPS-stimulated RAW264.7 cells. *K*-means clustering analysis showed that the 4-OI treatment and Tet2-knockout (KO) groups displayed similar expression patterns upon LPS stimulation, indicating that 4-OI suppresses LPS-induced genes by targeting TET2. Furthermore, they found TET2 deletion completely abolished the effect of exogenous itaconate on suppressing LPS-induced chemokine and cytokine genes, providing further evidence that TET2 is the target of itaconate. Additionally, itaconate targeted TET2 to suppress LPS-induced NF-κB and STAT1 signaling pathway genes. In an LPS-induced sepsis model, the researchers did not observe the anti-inflammatory effects of itaconate in TET2^−/−^ mice. These findings suggest that the IRG1–itaconate–TET2 axis plays a key physiological role in immune modulation. Notably, TET2 mutations can cause cancer such as acute myeloid leukemia. Whether itaconate inhibiting TET2 catalytic activity leads to cancer requires further investigation.

### Itaconate as a lysosomal inducer

Macrophages engulf invading bacteria through the endocytic or phagocytic pathway, and the bacteria are then delivered to the lysosomes to form the phagolysosome, ultimately leading to their destruction [94, 95]. Recently, Zhang et al. [96] revealed that itaconate regulated lysosomal activity during bacterial infection. The transcription factor EB (TFEB) can coordinate lysosomal biogenesis and regulate cellular responses to various stressors [50]. TFEB translocates from the cytosol to the nucleus to exert its function. Itaconate was found to induce TFEB nuclear translocation and activate TFEB through alkylating TFEB at Cys212. Itaconate and 4-OI promote lysosomal biogenesis clearance of bacteria by alkylating TFEB at Cys212. Finally, 4-OI was found to promote TFEB nuclear translocation and lysosomal biogenesis in human macrophages. These results indicates that 4-OI could be a lysosomal inducer to exert an anti-inflammatory role.

### Itaconate regulates programmed cell death 1 ligand 1

Programmed cell death 1 (PD-1) and its ligand (PD-L1), also known as cluster of differentiation 274 (CD274), are primarily expressed on the surface of antigen-presenting cells [97]. PD-1 binding to PD-L1 can conduct inhibitory signals and reduce the proliferation of CD8^+^ T cells in lymph nodes [98]. However, tumor cells exploit this mechanism by expressing PD-1 or PD-L1, leading to immune escape [99, 100]. PD-L1 is reportedly highly expressed in certain cell types in sepsis. Zhang et al. [101] demonstrated that PD-1 was highly expressed on T lymphocytes and PD-L1 was considerably upregulated on monocytes in patients with septic shock. A prospective study revealed that the high expression of PD-L1 in monocytes was closely related to the severity of the disease [102]. Meanwhile, multivariate analysis showed that PD-L1 expression in monocytes was an independent factor for high 28-day mortality in patients with septic shock. PD-L1 blockade exerts a protective effect on sepsis by inhibiting lymphocyte apoptosis and reversing monocyte dysfunction. Zhang et al. [103] reported that anti-PD-L1 antibody administration exerted a protective effect on cecal ligation and puncture (CLP)-induced sepsis in mice, indicating that anti-PD-L1 antibody treatment could be a promising strategy for immunosuppression-caused sepsis. In our study, we also found that anti-PD-L1 antibody treatment reduced mice mortality in CLP-induced sepsis [104]. In vitro, genome-wide expression analysis showed that 4-OI treatment significantly enhanced CD274 expression under LPS stimulus in RAW264.7 cells. Enrichment analysis revealed a strong negative correlation between Nrf2 and PD-L1, up to −97.6%, suggesting that 4-OI could regulate CD274 expression via Nrf2. Additionally, 4-OI inhibited the expression of PD-L1, and Nrf2 knockout significantly increased the expression level of PD-L1, indicating that 4-OI negatively regulated PD-L1 by activating Nrf2. Finally, Nrf2 ChIP-sequencing (ChIP-seq) analysis showed that the Nrf2 gene had three binding sites within intron 6 of the PD-L1 gene. The mutation of these binding sites significantly increased PD-L1 expression. Overall, 4-OI negatively regulated PD-L1 through Nrf2 binding to the PD-L1 gene to exert anti-inflammation in sepsis. This suggests that IRG1 may be used as an immunotherapeutic target in some diseases.

### The roles of itaconate in ferroptosis

Ferroptosis is a new type of iron-dependent programmed cell death. It is caused by the accumulation of iron-dependent lipid peroxides, which was first proposed in 2012 [105, 106]. The main mechanism of ferroptosis is that under the action of divalent iron or ester oxygenase, the unsaturated fatty acids, which are highly expressed on the cell membrane, are catalyzed to peroxidation of lipid, thus inducing cell death [107, 108]. In addition, it also showed a decrease of glutathione peroxidase 4 (GPX4), the regulatory core enzyme of antioxidant system [109]. Ferroptosis plays an important role in the occurrence and development of various diseases, especially in neurodegenerative diseases and ischemia–reperfusion (I/R) injury [110, 111].

The effects of GPX4 against ferroptosis is accomplished with the assistance of glutamate cysteine ligase (GCL). Studies showed that 4-OI significantly increased GCLM levels and reversed the decrease of LPS-induced GCLM levels [110]. Nrf2 is a major regulator of the antioxidant response. Studies have shown that activation of the Keap1–Nrf2 pathway significantly inhibits ferroptosis and alleviated disease severity [113]. By analyzing the STRING database and using Cytoscape 3.7.1 software, Song et al. [21] found that Nrf2 could directly or indirectly regulate ferroptosis-related proteins. In the protein–protein interaction network, they revealed that Nrf2 regulated ferroptosis mainly by affecting the synthesis and function of GPX4 and the peroxisome proliferator-activated receptor γ pathway, suggesting its role in directly or indirectly regulating the GPX4 expression. Another important target of Nrf2 is SLC7A11, which is responsible for GSH synthesis. Nrf2 binds directly to the ARE sequence of the SLC7A11 subunit promoter, thereby promoting SLC7A11 expression [114]. Overexpression of Nrf2 or knockdown of Keap1 increases the expression of SLC7A11, while inhibition of Nrf2 expression or overexpression of Keap1 decreases SLC7A11 protein expression [112].

In in vivo experiments, sepsis-induced acute lung injury (ALI) symptoms were more severe in Nrf2^−/−^ mice, and the protective effect of 4-OI was lost in Nrf2^−/−^ mice. Mechanistically, He et al. [112] found that 4-OI protected mice from ALI by inhibiting ferroptosis through upregulating Nrf2. Interestingly, they observed that 4-OI reduced tissue iron levels in Nrf2^−/−^ mice, suggesting that 4-OI may regulate intracellular iron levels through an Nrf2-independent mechanism. Therefore, regulating the Nrf2 pathway is a potential treatment option for ferroptosis-related diseases. In addition, activated M1 macrophages are not susceptible to ferroptosis, indicating that itaconate may regulate ferroptosis through its effects on macrophage polarization [115]. Overall, these studies demonstrate that itaconate has both exerted anti-inflammatory and inhibitory effects on ferroptosis; both of these effects may serve as protective mechanisms in vivo.

### The roles of itaconate in pyroptosis

Pyroptosis was first proposed by Cookson et al. in 2001 to describe the caspase-1-dependent cell death pattern found in macrophages [116]. Pyroptosis is mainly regulated by caspase-1, caspase-4, caspase-5, and caspase-11 signaling pathways [117]. In the caspase-1-dependent signaling pathway, pathogen-associated molecular patterns (PAMPs) or host-derived danger signal molecules (DAMPs) activate NLRP3 and caspase-1. Caspase-1 will directly lyse gasdermin D (GSDMD) to form GSDMD-N, and GSDMD-N forms a pore in the host cell membrane to regulate the release of cytosolic contents. In addition, caspase-1 can cleave pro-IL-1β and pro-IL-18 precursor to IL-1β and IL-18.

Hooftman et al. [26] used LPS and nigericin to induce pyroptosis of BMDM in vitro and found that itaconate and its derivative 4-OI inhibited the activation of NLRP3 inflammasome, caspase1, IL-1β, and IL-18. Mechanically, this was related to the inhibition of the NLRP3-NEK7D interaction by 4-OI. In vivo, the results showed that 4-OI reduced NLRP3-driven peritonitis and inhibited IL-1β release from peripheral blood mononuclear cells isolated from patients with cryopyrin-associated periodic syndrome. In addition, Bambouskova et al. [118] reported that itaconate interfered with the signal 2 events of NLRP3 activation and inhibited the cleavage of IL-1β and GSDMD. A recent study showed that itaconate and its derivatives could be used to treat congenital ISG15 deficiency by reducing apoptosis and pyroptosis [119]. Treatment with itaconate reduced caspase-1 activity in ISG15^−/−^ macrophages stimulated with IFN-α. Furthermore, our study showed that IRG1/itaconate attenuated concanavalin A-induced acute liver injury in vivo by inhibiting pyroptosis [120]. Collectively, these results indicate that itaconate can alleviate certain disease symptoms by inhibiting pyroptosis.

### The roles of itaconate in epigenetic landscape

Epigenetics refers to heritable changes in gene expression, despite no changes in DNA sequence, such as DNA methylation, histone modifications, and noncoding RNA regulation. Recently, Aso et al. [121] reported itaconate inhibited Th17 cell differentiation and promoted Treg cell differentiation to ameliorate autoimmunity by epigenetic reprogramming. Itaconate could inhibit methionine adenosyltransferase and IDH1/2 enzymatic activity to induce histone demethylation while altering the accessibility of transcription factors to certain genomic regions. Finally, they found itaconate changed chromatin accessibility of essential transcription factors at the Il17a and Foxp3 loci. In addition, Domínguez-Andrés et al. [122] reported that itaconate influenced the histone 3 lysine 27 acetylation, suggesting itaconate could exert its effects through epigenetic mechanisms. Future studies are warranted to fully investigate the roles of itaconate in epigenetic landscape.

### The effect of itaconate in anti-inflammation

#### Sepsis

Sepsis is a systemic inflammatory response syndrome caused by an imbalance of immune response and multiple organ dysfunction, which is the result of an infection [123]. It can cause inflammatory storms and immunosuppression, leading to multiple organ failure. Reportedly, 4-OI significantly increased survival and prevented LPS-induced lethality [112, 124]. In vitro, 4-OI inhibited the production of inflammatory cytokines induced by LPS in RAW264.7 and BMDM cells. Mechanistically, Zhang et al. [125] reported that 4-OI inhibited ROS production by activating Nrf2/HO-1 and subsequently inhibited inflammation in mice. Another study reported that 4-OI played an anti-inflammatory role by promoting the alkylation of GAPDH and inhibiting glycolysis in mice [64]. Mills revealed that dimethyl malonate, an inhibitor of succinate oxidation, protected mice from LPS lethality in mice [126]. As previously described, itaconate acts as an SDH inhibitor; therefore, it may prevent lethality in sepsis as well through inhibition of succinate oxidation. Nevertheless, further experiments are needed to verify this hypothesis.

The aforementioned studies showed that 4-OI significantly reduced the severity of LPS-induced sepsis. Moreover, the CLP-induced sepsis model is commonly used in drug and target research. Interestingly, Wu et al. [127] reported that IRG1 depletion increased the survival rate of mice in CLP-induced sepsis and ameliorated the severity of sepsis. Furthermore, sequencing studies and itaconate rescue experiments revealed that IRG1-mediated inflammatory responses could occur in an itaconate-independent mode. This discrepancy may be caused by differences in the models used or the dual roles of innate immunity in regulating inflammation with different infections. Therefore, appropriate inflammation is beneficial for pathogen elimination, whereas unrestricted activation of innate immunity may lead to cytokine storms and worsen sepsis. IRG1 deletion may reduce the excessive immune response to external stimuli in CLP-induced sepsis.

**Fig. 4:**
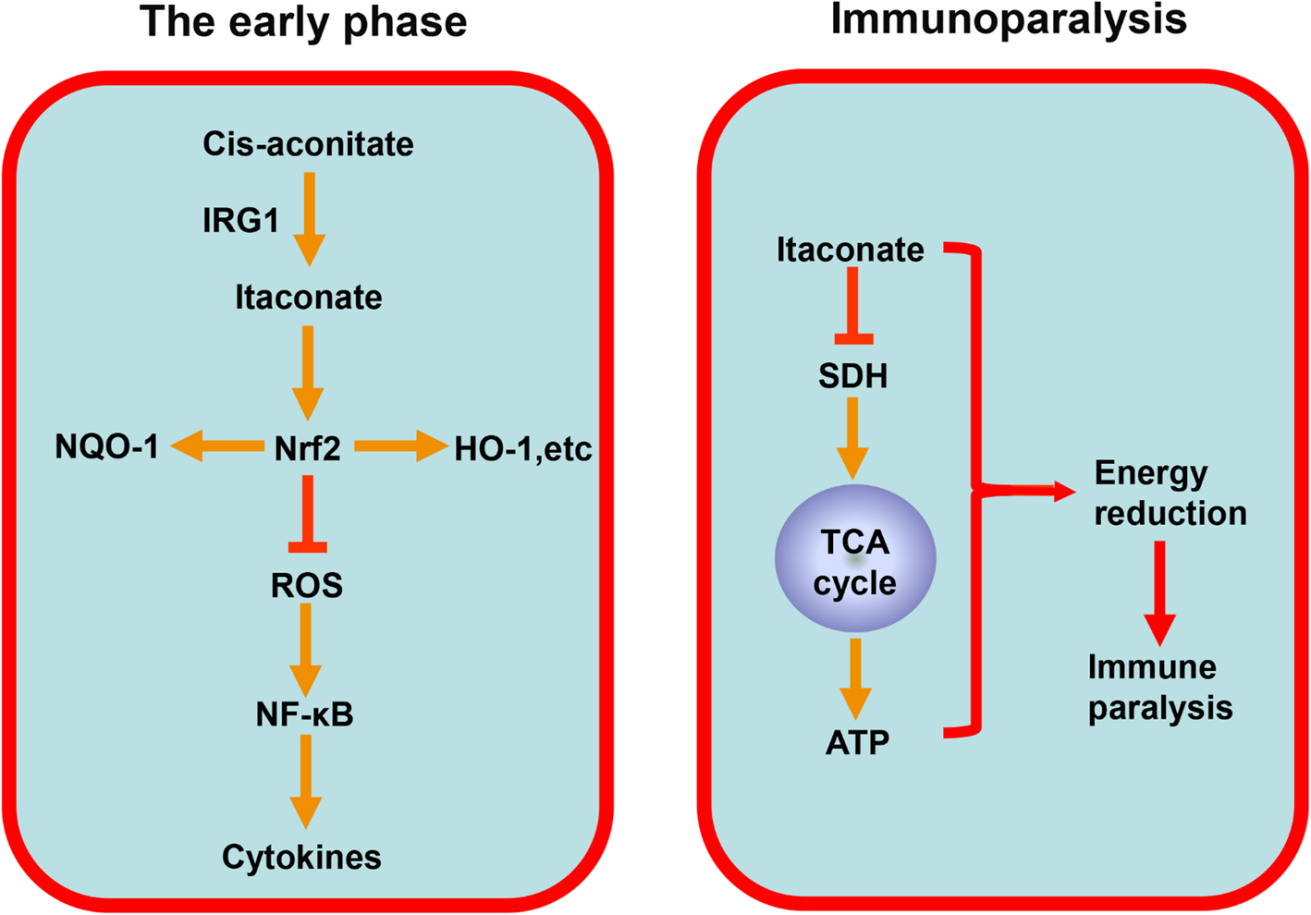
The role of itaconate in sepsis

As the disease progresses, the heightened inflammatory state transitions into immunosuppression, increasing susceptibility to secondary infections. Sepsis induces a state of immunocompromise in the host during the late stages, known as sepsis-associated immunosuppression (SAIS). In clinical practice, SAIS often results in the death of patients with sepsis due to secondary infections, characterized by neutrophil paralysis and monocyte paralysis. Meanwhile, Li et al. [44] demonstrated elevated expression of IRG1 in peripheral blood mononuclear cells from patients with sepsis and LPS-tolerized mouse macrophages. Furthermore, they found that IRG1 knockdown by small interfering RNA did not affect TLR-induced production of TNF-α, IL-6, and IFN-β in WT macrophages but significantly increased the production of these cytokines in LPS-tolerized macrophages. Zhu et al. [128] reported that itaconate inhibited SDH and its anabolic role in mitochondrial ATP production. This inhibition led to TCA cycle isolation at the late stage of sepsis, ultimately resulting in decreased ATP levels. Furthermore, they revealed dichloroacetate could inhibit itaconate production in septic mice, increase TCA cycle activity, reverse septic shock, and restore innate and adaptive immunity as well as organ function. Hence, the proinflammatory effect of itaconate in the late stage may be attributed to the inhibition of the TCA cycle and reduction of energy production. Moreover, Dominguez-Andres et al. [122] reported that β-glucan increased the expression of SDH and suppressed IRG1 expression in human monocytes, thereby contributing to TCA cycle integrity and restoring immune function. Therefore, appropriate inhibition of itaconate production during immunoparalysis phase may help control sepsis progression (Fig. 4).

#### Inflammatory bowel disease

IBD is a nonspecific chronic intestinal inflammatory disease of unknown etiology, encompassing ulcerative colitis and Crohn’s disease. Activation of the MAPK/NF-κB pathway is an important therapeutic factor in a variety of inflammatory diseases. Patients with IBD exhibited increased activation of NF-κB in mucosal macrophages and epithelial cells, leading to the production of TNF-α and other proinflammatory factors, and exacerbating the inflammatory response [129]. The JAK/STAT signaling pathway also plays an important role in the pathogenesis of IBD. Reportedly, STAT3 and p-STAT3 expression significantly increased in patents with ulcerative colitis and Crohn’s disease [130]. Furthermore, STAT3 in acquired immune cells contributes to the pathogenesis of IBD. Wang et al. [131] reported that DI decreased the high inflammatory state of ulcerative colitis and reduced the risk of colitis-associated cancer in mice. Kim et al. [132] demonstrated that IRG1 deletion increased inflammatory cytokine and chemokine levels, resulting in severe dextran sulfate sodium (DSS)-induced colitis in mice. And 4-OI alleviated DSS-induced colitis in IRG1^−/−^ mice and decreased the expression of inflammatory cytokines and chemokines. Our study also showed that 4-OI alleviated DSS-induced colitis in mice by activating the Nrf2/HO-1 pathway, inhibiting ROS-mediated NF-κB pathway and suppressing pyroptosis [133]. Taken together, these studies indicated IRG1/itaconate may be a potential therapeutic target for IBD treatment.

### The effect of itaconate in antioxidation

#### Ischemia–reperfusion injury

After restoring blood flow on the basis of ischemia, the injury of tissues and organs worsens instead, which is called ischemia–reperfusion (I/R) injury [134]. In 2016, Lampropoulou et al. [58] first reported that itaconate modulated macrophage metabolism by inhibiting SDH-mediated succinate oxidation to relieve myocardial I/R injury in mice. Subsequently, Cordes et al. [56] demonstrated that the addition of itaconate to reperfusion fluids after mouse cerebral I/R injury markedly increased glutathione levels and ROS/nitrogen species to improve neurological function. These findings suggest that itaconate could minimize ROS and tissue damage during reperfusion by inhibiting SDH activity.

Zhang et al. [135] reported preoperative aerobic exercise induced the release of high-mobility group box 1 in the TCA cycle, affecting Kupffer cells in an Nrf2-dependent manner and significantly alleviating liver injury caused by I/R injury in mice. Additionally, Yi et al. [136] demonstrated that IRG1/itaconate protected against hepatic I/R injury by activating the Nrf2 pathway in mice. They also found that liver damage was higher in Nrf2^−/−^ mice subjected to I/R injury, and 4-OI treatment alleviated liver injury. In addition, studies have reported that itaconate and its derivatives exert protective effects in cerebral ischemia/reperfusion injury. DMI significantly inhibited the toxic conversion of the peri-infarct microglia and IL-1β levels in cardiac I/R injury in mice [137]. Vigil et al. [138] found that endogenous Acod1 is protective in cerebral I/R injury in mice. However, the specific mechanism of itaconate is not further explored in these studies, though the inhibitory effect on the release of inflammatory factors was likely related to the activation of Nrf2. Thus, itaconate and 4-OI protect against I/R injury mainly by activating the Nrf2 pathway and inhibiting SDH activity.

#### The effect of itaconate in immunomodulatory diseases

Systemic lupus erythematosus (SLE) is an autoimmune disease that affects multiple systems and organs of the body, characterized by complex clinical manifestations and a prolonged and recurrent course. Currently, the drug treatment for SLE mainly includes nonsteroidal anti-inflammatory drugs, antimalarial drugs, glucocorticoids, and immunosuppressants [139]. Tang et al. [140] reported significant Nrf2 activation in SLE patient-derived THP-1 macrophages upon treatment with 4-OI. They found that pretreatment with 4-OI significantly inhibited the production of TNF-α, IL-1β, and IL-6 in SLE patient-derived peripheral blood mononuclear cells (PBMCs). This effect was attenuated when Nrf2 was silenced or knocked out. Recently, Blanco et al. [92] investigated the role of itaconate derivative in the treatment of established murine lupus in F1 lupus-prone mice. They found 4-OI treatment decreased the severity of lupus nephritis and levels of autoantibodies. Use of 4-OI improved thrombocytopenia and modulated lymphoid organ responses. Furthermore, 4-OI administration reduced various antinuclear antibodies and dysregulation of the type I IFN pathway. Autoantibody ICs can trigger type I IFN production, which contributes to immune dysregulation. Mechanistically, they hypothesized 4-OI inhibited IC formation by decreasing autoantigen generation and subsequently decreasing immune dysregulation. Additionally, the suppression of inflammatory cytokines released by 4-OI treatment may also inhibit autoantibody generation. Jing et al. [141] reported that inhibiting glycolysis in macrophages improved lupus nephritis in mice. Considering itaconate is a glycolysis inhibitor, it may relieve SLE through glycolysis inhibition as suggested by Blanco et al. [92]. Furthermore, 4-OI was found to reduce the mitochondrial antiviral signaling protein (MAVS) oligomerization in mice. ROS plays a role in MAVS dependent responses in SLE. Therefore, 4-OI may inhibit ROS production through the Nrf2 pathway, leading to a decrease in MAVS oligomerization. Hedrich et al. [142] reported that the JAK/STAT pathway was involved in the pathogenesis of SLE. In vivo, 4-OI was shown to inhibit JAK1 activation. Hence, 4-OI may alleviate the severity of SLE through multiple signaling pathways. Nevertheless, further experiments are necessary to prove the safety and feasibility of 4-OI for SLE treatment in the future.

#### Multiple sclerosis

Multiple sclerosis (MS) is an autoimmune disease that specifically targets the white matter of the central nervous system, causing its demyelination. DMF is currently an approved drug for the treatment of relapsing MS. DI, an analog of DMF, may also have therapeutic effects in MS. However, reports on the treatment of MS with itaconate or its derivatives are limited. Kuo et al. [75] reported that DI ameliorated the severity of experimental autoimmune encephalomyelitis in mice models. Further analysis revealed that DI inhibited peripheral Th1/Th17 differentiation and enhanced Nrf2 and HO-1 expression. Itaconate inhibits SDH activity, which in turn inhibit HIF-1α activation and IL-1β production, ultimately obstructing Th17 differentiation. Therefore, they hypothesized that the activation of Nrf2/HO-1 pathway and inhibition of SDH/HIF-1α activity were the primary protective mechanisms. Additionally, Hoyle et al. [143] reported that DI inhibited NLRP3 activation in microglia and macrophages and directly inhibited GSDMD lysis, thus alleviating MS. Kornberg et al. [63] reported that DMF alleviated the severity of MS by targeting GAPDH and aerobic glycolysis. As an analog of DMF, further exploration is needed to determine whether DI has the same mechanism of action in MS.

#### Other diseases

Cryopyrin-associated periodic syndrome (CAPS) is a group of autoinflammatory diseases characterized by autosomal dominant mutations in NLRP3 [144]. Inhibiting NLRP3 inflammasome formation could be a potential treatment for NLRP3-driven diseases [145]. Itaconate abolished NLRP3–NEK7 connection and “dicarboxypropylated” C548 on NLRP3 in a study, suggesting that itaconate might play a role in the treatment of NLRP3-driven diseases [26]. Song et al. [146] reported that 4-OI prevented abdominal aortic aneurysm (AAA) formation by enabling Nrf2 in mice models. Zhao et al. [147] demonstrated that DI protected against LPS-induced mastitis in mice through Nrf2 activation and NF-κB signaling pathways inhibition. Meanwhile, Xu et al. [148] revealed that DI protected against LPS-induced endometritis through Nrf2/HO-1 signaling pathway activation in mice models. Daly et al. [149] reported that increasing itaconate in plasma was related to improved rheumatic activity scores in patients with rheumatoid arthritis receiving antirheumatic drug treatment, suggesting that itaconate might exert a protective effect in rheumatoid arthritis. Tian et al. [150] reported that 4-OI protected against renal fibrosis by Nrf2 activation and NF-κB signaling pathways inhibition in mice models. Sun et al. [151] reported that 4-OI inhibited osteoclastogenesis by activating Nrf2 signaling in vitro and in vivo. Recently, Xin et al. [152] reported that 4-OI ameliorated periodontal destruction through the disassociation of KEAP1-Nrf2 and Nrf2 activation in mice models. A recent study published in *Obesity* showed that IRG1/itaconate played an important role in decreasing obesity risk and insulin resistance [153]. Meanwhile, DMT ameliorated cognitive impairment induced by a high-fat diet in mice [154]. These results indicate that itaconate has a promising application prospect in the treatment of different diseases (Table 1). Further evaluation is needed to determine the therapeutic effect and mechanism of itaconate and its derivatives in disease.

**Table 1:** The participation mechanisms of itaconate and derivatives in different disease models

Disease	Model	Species	Agents	Mechanisms	References
Sepsis	LPS-induced lethality	Mice	4-OI	Nrf2/HO-1	Zhang et al.
	LPS-induced lethality	Mice	4-OI	GAPDH	Liao et al.
	CLP-induced sepsis	Mice	4-OI	Nrf2–PDL1	Zhang et al.
	CLP-induced sepsis	Mice	IRG1 deletion	CDK2–IRG1	Wu et al.
IBD	DSS-induced colitis	Mice	DI	Inflammatory cytokine	Wang et al.
	DSS-induced colitis	Mice	4-OI	Inflammatory cytokine	Kim et al.
	DSS-induced colitis	Mice	4-OI	Nrf2/HO-1, pyroptosis	Yang et al.
I/R injury	Myocardial I/R injury	Mice	DI	SDH	Lampropoulou et al.
	Cerebral I/R injury	Mice	Exogenous itaconate	SDH, Nrf2/ROS	Cordes et al.
	Hepatic I/R injury	Mice	Exercise	Nrf2/HO-1	Zhang et al.
	Hepatic I/R injury	Mice	4-OI	Nrf2/HO-1	Yi et al.
	Hepatic I/R injury	Mice	DI	NLRP3	Ma et al.
SLE	Cells from SLE patients	Human	4-OI	Nrf2/HO-1	Tang et al.
	Murine lupus	Mice	4-OI	Keap1/Nrf2, Glycolysis	Blanco et al.
MS	EAE	Mice	DI	Nrf2/HO-1, SDH	Kuo et al.
	Mixed glia	Mice, human	DI, 4-OI	NLRP3	Hoyle et al.
CAPS	Cells from CAPS patients	Human	4-OI	NLRP3	Hooftman et al.
AAA	Ang II-induced AAA	Mice, human	4-OI	Nrf2	Song et al.
Mastitis	LPS-induced mastitis	Mice	DI	Nrf2	Zhao et al.
Endometritis	LPS-induced endometritis	Mice	DI	Nrf2/HO-1	Xu et al.
Renal fibrosis	Adenine-induced fibrosis	Rats	4-OI	Nrf2	Tian et al.
Osteoclastogenesis	RANKL-induced osteoclastogenesis	Mice	4-OI	Nrf2	Sun et al.
Periodontitis	Experimental periodontitis	Mice	4-OI	Keap1/Nrf2	Xin et al.
Myogenesis	BaCl2-induced muscle injury	Mice	4-OI	SDH	Oh et al.

#### Cancer

Recently, several studies reported that itaconate plays a vital role in cancer immunometabolism. Wang et al. [131] reported that DI protected against colitis-associated colorectal cancer by inhibiting the IL-1β secretion and macrophages infiltration into the tumor microenvironment, thereby alleviating the high inflammatory state of colitis and reducing the risk of colitis-associated colorectal cancer development. However, itaconate can affect the metabolic changes in macrophages, which may contribute to cancer development. A significant increase in itaconate levels was found in peritoneal tissue-resident macrophages (pResMϕ) of peritoneal tumor-bearing mice [155]. Itaconate reduced oxidative phosphorylation (OXPHOS) and OXPHOS-driven ROS production in pResMϕ, thereby inhibiting ROS-mediated MAPK activation in the tumor. Decreased ROS levels and MAPK inhibition subsequently promoted tumor growth. This suggests that peritoneal tumors affect macrophage metabolism, and, in turn, the changes in macrophage metabolism promote the progression of malignant tumors through a certain mechanism. Additionally, they found significantly elevated levels of IRG1 in monocytes isolated from ascites fluid of patients with ovarian carcinoma. Similarly, Pan et al. [156] reported that IRG1 was upregulated in human glioma cell lines and clinical specimens, and patients with high IRG1 expression had a worse prognosis. Recently, a study published in *Nature Metabolism* revealed that itaconate secreted by myeloid-derived suppressor cells suppressed the cytotoxic CD8^+^ T cell proliferation by inhibiting the biosynthesis of aspartate and serine/glycine, thereby promoting tumor growth [157]. In addition, targeting IRG1 reversed the immunosuppressive function of tumor-associated macrophages and enhanced cancer immunotherapy [158]. Liu et al. [159] showed that 4-OI triggered ferritinophagy-dependent ferroptosis, thereby inhibiting the growth of human retinoblastoma cells, while Zhao et al. reported that neutrophils promoted breast cancer metastasis by resisting ferroptosis through IRG1 [160]. Another study has demonstrated that DI suppresses thymic carcinoma cell growth and promotes apoptosis by targeting the LDHA–mTOR axis, thus playing an anticancer role [161]. Gautam et al. [162] reported DI is an anticancer drug for diethylnitrosamine-induced hepatocellular carcinoma in albino Wistar rats. Recently, we found 4-OI inhibited aerobic glycolysis by targeting GAPDH to promote cuproptosis in colorectal cancer in mice models [163]. These studies indicate that itaconate and its derivatives have different effects on different tumors, which can either inhibit the growth of some tumors or promote the growth of others (Table 2). The role of itaconate and its derivatives in cancer requires to be further exploration.

**Table 2:** The role of itaconate and derivatives in cancer

Disease	Model	Agents	Mechanisms	Role	Reference
Colorectal cancer	DSS-induce mice model	DI	Inflammatory cytokine	Antitumor effect	Wang et al.
Peritoneal tumors	Peritoneal tumors-bearing mice	IRG1 deletion	OXPHOS, ROS, MAPK	Prompt tumor growth	Weiss et al.
Glioma	Xenograft transplantation model	IRG1 knockdown	Cell cycle regulatory proteins	Prompt tumor growth	Pan et al.
Retinoblastoma	Xenograft mouse models	4-OI	Ferroptosis	Antitumor effect	Liu et al.
Thymic carcinoma	Cell line Ty82	DI	LDHA-mTOR axis	Antitumor effect	Hayashi et al.
Hepatocellular carcinoma	Diethylnitrosamine-induced HCC	DI	Mitochondrial apoptosis	Antitumor effect	Gautam et al.
Breast cancer	Allografted tumors	IRG1 deletion	CD8^+^ T cells	Prompt tumor growth	Zhao et al.

#### The antibacterial and antiviral effects of itaconate

Since 1977, scientists have revealed that itaconate inhibits the growth of *Pseudomonas indigo* under glucose deprivation. Itaconate plays an antibacterial role mainly by inhibiting the activity of isocitrate lyase in bacteria, thus blocking the glyoxylate shunting pathway necessary for bacterial growth and pathogenicity. Furthermore, itaconyl-coenzyme A, an intermediate metabolite of itaconate, can inhibit the activity of B12-dependent methyl malonyl-CoA mutase, thereby impeding bacterial growth, such as *M. tuberculosis.*. Tuberculosis is a prevalent and deadly infectious disease caused by *M. tuberculosis*. Nair et al. [164] found that IRG1^−/−^ mice treated with *M. tuberculosis* had a higher mortality rate than that of WT mice, indicating IRG1/itaconate inhibited immunopathology in *M. tuberculosis* infections. RNA sequencing analyses showed that IRG1/itaconate inhibited *M. tuberculosis*-induced inflammation in myeloid cells at the transcriptional level. Ruetz et al. [165] reported that itaconyl-coenzyme A inhibited the activity of *M. tuberculosis* through inhibiting B12-dependent methylmalonyl-CoA mutase, an important mutase for *M. tuberculosis*. In addition, Naujoks et al. [39] reported that itaconate modified the proteome of *Legionella pneumophila*-containing vacuoles. Thus, itaconate restricts bacterial growth mainly by inhibiting isocitrate lyase, a key enzyme for bacterial growth, or by inhibiting propionyl-CoA carboxylase.

Moreover, several studies have reported that itaconate has an inhibitory effect on virus growth. Daniels et al. [52] reported that itaconate inhibited Zika virus growth, and Sohail et al. [166] found that 4-OI inhibited influenza A virus transcription in mononuclear cells. Liu et al. [167] reported an association between polymorphisms in the *IRG1* gene and the immune response to hepatitis B vaccination in human. Furthermore, they found that IRG1 inhibited the HBV life cycle, indicating that IRG1 may exert some effects in the treatment of hepatitis B. Song et al. [168] first reported that the expression of itaconate in patients with coronavirus disease 2019 (COVID-19) decreased progressively with the severity of COVID-19 in humans. Olagnier et al. [78] found that 4-OI decreased the release of progeny virus particles in cells infected with SARS-CoV-2 in humans.

#### Perspective and conclusions

**Fig. 5:**
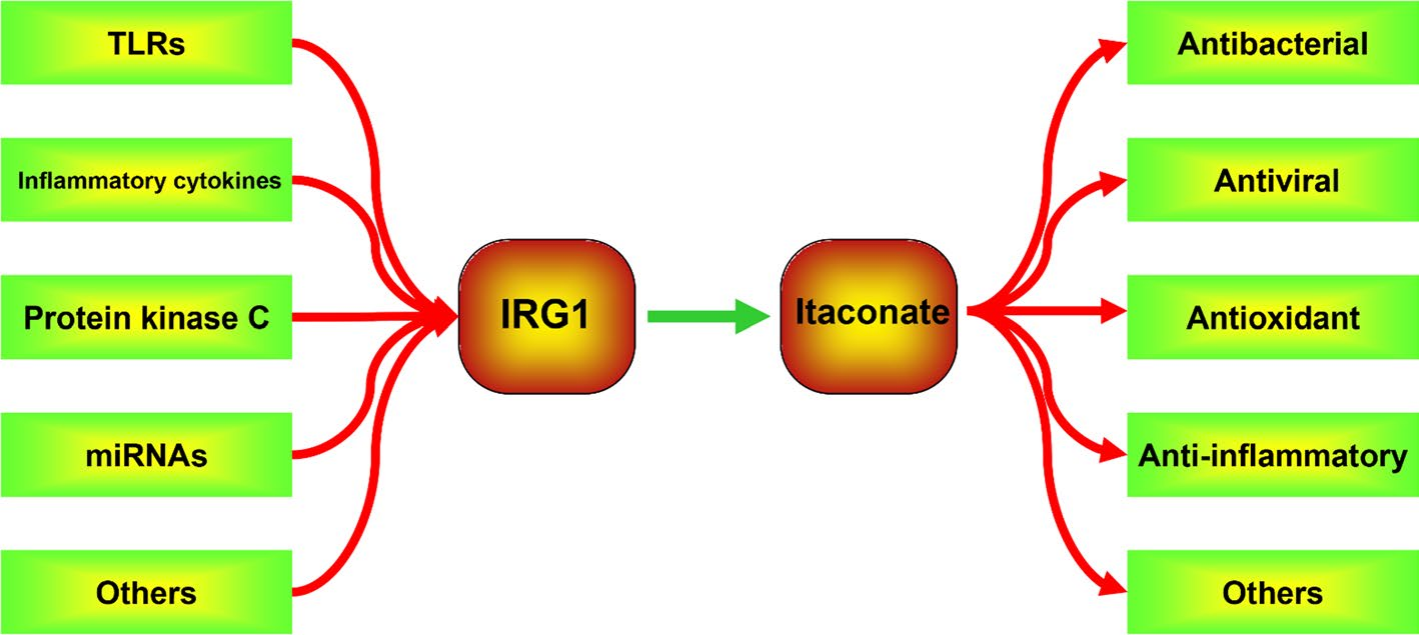
The modulation and role of IRG1/itaconate

As an intermediate metabolite in the TCA cycle, itaconate regulates the interaction between metabolism, immunity, and inflammation, providing alternative therapeutic strategies for treating immune-inflammatory diseases (Fig. 5). Currently, the anti-inflammatory and antioxidant effects of itaconate have been demonstrated in animal and in vitro experiments, and DMF has been used in clinical treatment for MS. Itaconate, as an endogenous metabolite, is a critical regulator of immune modulation with low toxicity, and it possibly could have clinical applicability pending long-term safety studies. The anti-inflammatory effect of itaconate is similar to the discovery of the antibiotic effects of microbial metabolites, which could contribute to the exploration of anti-inflammatory therapies. In addition, excessive immune suppression can lead to immune paralysis and reduce body resistance; thus, the effect of itaconate on tumor growth should not be overlooked. Presently, reports on the effect of itaconate on tumors are conflicting. Itaconate may play different roles in different malignant tumors, necessitating further investigation. Overall, in this study, we provide systematic introduction to the discovery and structure of itaconate, its biosynthesis and metabolism, itaconate derivatives, the signaling pathway of itaconate action, and its therapeutic potential in various diseases. Much remains to be learned this metabolite, and we anticipate that itaconate will play a role in the future treatment of inflammatory diseases.

## Data Availability

Not applicable.

## Abbreviations

4-EI: 4-Ethyl itaconate
4-OI: 4-Octyl itaconate
AAA: Abdominal aortic aneurysm
ALDOA: Aldolase A
ALI: Acute lung injury
ARE: Antioxidant response element
ATF3: Activating transcription factor 3
BMDCs: Bone marrow-derived dendritic cells
BMDMs: Bone marrow-derived macrophages
BoNT/A: Botulinum neurotoxin type A
CAD: Cis-aconitate decarboxylase
CAPS: Cryopyrin-associated periodic syndrome
CD274: Cluster of differentiation 274
cGAS: Cyclic GMP–AMP synthase
CLP: Cecal ligation and puncture
DAMPs: Danger signal molecules
DI: Dimethyl itaconate
DMF: Dimethyl fumarate
GAPDH: Glyceraldehyde-3-phosphate dehydrogenase
GCL: Glutamate cysteine ligase
GPX4: Glutathione peroxidase 4
GSDMD: Gasdermin D
HIF-1α: Hypoxia-inducible factor-1α
IBD: Inflammatory bowel disease
IFNβ: Interferon beta
IFN-γ: Interferon-gamma
IKK: IκB kinase
I/R: Ischemia–reperfusion
*IRG1*: Immune responsive gene 1
JAK1: Janus kinase 1
LTA: Lipoteichoic acid
MAVS: Mitochondrial antiviral signaling
mRNA: Messenger RNA
mtROS: Mitochondrial reactive oxygen species
MS: Multiple sclerosis
MYD88: Myeloid differentiation primary response 88
NF-κB: Nuclear factor-kappaB
NQO1: NAD(P)H:quinone oxidoreductase 1
Nrf2: Nuclear factor E2-related factor 2
OXPHOS: Oxidative phosphorylation
PAMPs: Pathogen-associated molecular patterns
PBMCs: Peripheral blood mononuclear cells
PD-L1: Programmed cell death 1 ligand 1
pResMϕ: Peritoneal tissue-resident macrophages
PKC: Protein kinase C
ROS: Reactive oxygen species
SAIS: Sepsis-associated immunosuppression
SDH: Succinate dehydrogenase
SLE: Systemic lupus erythematosus
STING: Stimulator of interferon genes
TBK1: TANK binding kinase 1
TCA: Tricarboxylic acid
TET2: Ten-Eleven Translocation-2
TFEB: Transcription factor EB
TLRs: Toll-like receptors
